# Application of spectral CT in diagnosis, classification and prognostic monitoring of gastrointestinal cancers: progress, limitations and prospects

**DOI:** 10.3389/fmolb.2023.1284549

**Published:** 2023-10-25

**Authors:** Yuqin Hong, Lijuan Zhong, Xue Lv, Qiao Liu, Langzhou Fu, Daiquan Zhou, Na Yu

**Affiliations:** ^1^ Department of Radiology, The Third Affiliated Hospital of Chongqing Medical University (Gener Hospital), Chongqing, China; ^2^ Department of Radiology, The People’s Hospital of Leshan, Leshan, China

**Keywords:** gastrointestinal cancers, spectral CT, multi-parameter imaging, diagnosis, efficacy evaluation

## Abstract

Gastrointestinal (GI) cancer is the leading cause of cancer-related deaths worldwide. Computed tomography (CT) is an important auxiliary tool for the diagnosis, evaluation, and prognosis prediction of gastrointestinal tumors. Spectral CT is another major CT revolution after spiral CT and multidetector CT. Compared to traditional CT which only provides single-parameter anatomical diagnostic mode imaging, spectral CT can achieve multi-parameter imaging and provide a wealth of image information to optimize disease diagnosis. In recent years, with the rapid development and application of spectral CT, more and more studies on the application of spectral CT in the characterization of GI tumors have been published. For this review, we obtained a substantial volume of literature, focusing on spectral CT imaging of gastrointestinal cancers, including esophageal, stomach, colorectal, liver, and pancreatic cancers. We found that spectral CT can not only accurately stage gastrointestinal tumors before operation but also distinguish benign and malignant GI tumors with improved image quality, and effectively evaluate the therapeutic response and prognosis of the lesions. In addition, this paper also discusses the limitations and prospects of using spectral CT in GI cancer diagnosis and treatment.

## Introduction

Gastrointestinal (GI) cancers represent malignant tumors from the gastrointestinal tract and the accessory organs of digestion, including esophageal, gastric, colorectal, liver, and pancreatic cancers, and the colorectal, liver, and gastric cancers are the second to the fourth most predominant cause of cancer-related mortality worldwide according to the Global Cancer Statistics 2020 (Sung et al., 2021). A forecast of the global burden of cancer mortality and morbidity, by 2040, new cases of GI cancer and deaths will increase significantly according to CANCER TOMORROW (World Health Organization, 2022). Epidemiologic data indicate a significant increase in the incidence of colorectal cancer in younger populations in the past three decades. Moreover, recent evidence also demonstrates a similar trend in gastric, pancreatic, and biliary tract cancers (Ben-Aharon et al., 2023). The management of gastrointestinal cancer primarily encompasses surgical intervention, chemotherapy, radiotherapy, and targeted biological therapy, thereby facilitating the attainment of curative intent or optimal tumor control through their synergistic integration. Due to the early clinical symptoms of GI cancer are not typical and the degree of malignancy is high. Most patients diagnosed with digestive GI cancers have been in an advanced stage, especially pancreatic cancer. Their poor response to treatment highlights the necessity of early detection, early diagnosis, and early treatment, which further arises higher requirements for accurate staging of the disease before surgery besides evaluation of the efficacy of tumor treatment and prognosis. Endoscopy serves as the gold standard for diagnosing GI cancers; however, due to its invasive nature and limited exploratory capabilities, alternative examination methods are required for adjunctive diagnosis. Imaging examination is the most important non-invasive examination method for GI tumors, including computed tomography (CT), magnetic resonance imaging (MRI), positron emission tomography (PET), ultrasound (US), and other medical imaging techniques (Shigeyoshi et al., 2014; Schneider et al., 2016; Dimitrakopoulou-Strauss et al., 2017), especially CT is widely used for staging comparison and surgical evaluation before treatment.

**FIGURE 1:**
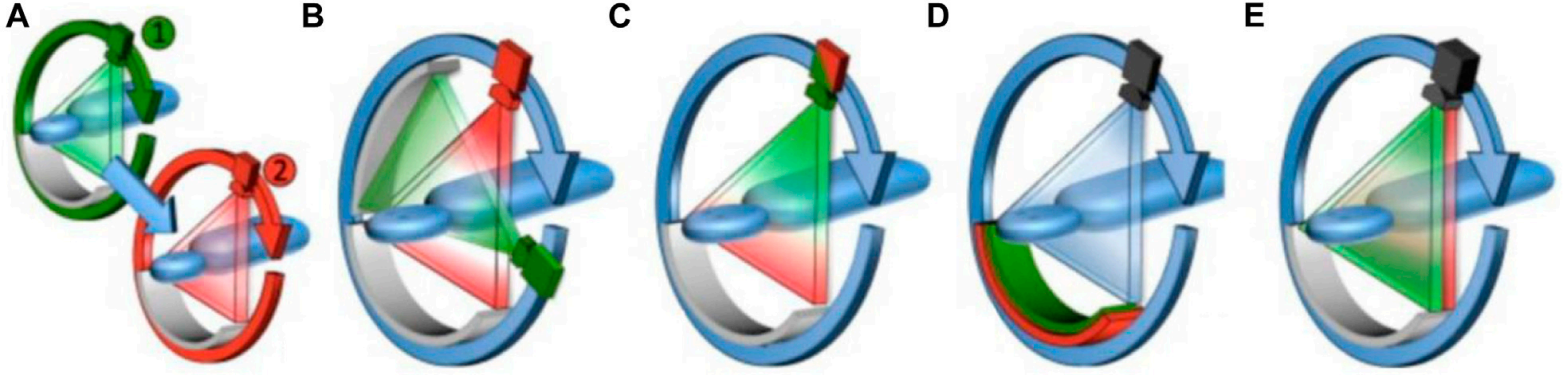
Schematic diagram of the operating principles for the different clinical dual-energy CT platforms currently available. **(A)**, Rotate–rotate CT, where sequential low- and high-energy acquisitions are obtained. **(B)**, Dual Source CT, where low- and high-energy data is acquired using two X-ray source and detector pairs. **(C)**, Rapid-kVp-switching CT, where the tube potential of a single X-ray source is rapidly switched between low- and high-energies. **(D)**, Multilayer detector CT, where the front layer of a sandwich detector preferentially absorbs low-energy X-ray photons, while the back layer absorbs the remaining high-energy X-ray photons. **(E)**, Split-beam CT, where a two-part filter mounted along the patient axis length modulates the X-ray spectra into a high- and low-energy spectrum for each half of the beam from a single non-switching source (Yeh et al., 2017).

CT is considered the greatest invention in medical imaging since Roentgen’s discovery of X-rays, which divides overlapping images of the human body into sectional images without structural overlays. However, traditional CT mainly provides anatomical and morphological evaluation of organs and tissues for GI cancers, but cannot accurately evaluates the benign, malignant lesions, and the true extent of the lesions. The emergence of spectral CT has opened a new chapter in a revolution, breaking away from the single-parameter anatomical diagnostic mode in CT imaging diagnosis and ushering in a new era of multi-parameter diagnosis (Marin et al., 2014). Spectral CT also called dual-source CT or dual-energy CT, was first hypothesized in the 1970s (Vetter et al., 1986), but it took decades for the first spectral CT to become available in the clinic in 2006 (Flohr et al., 2006). Since then, the evaluation and application of spectral CT have entered a stage of rapid development and have been technically mature until recent years. The main technique approaches currently for spectral imaging: are rotate-rotate CT (A), Dual Source CT (B), Rapid-kVp-switching CT (C), Multilayer detector CT (D), and Split-beam CT (E) (Figure 1) (Adam et al., 2021). Spectral CT can generate dual-energy data and obtain multiple groups of single-energy images to realize spatial energy spectrum analysis of data (McCollough et al., 2015). The clinical application of spectral CT is mainly realized through energy spectrum tools. Based on these energy spectrum tools, the original spectral CT data can be further information mining, multi-modal quantitative parameters can be obtained, and the composition difference of tissues and blood supply characteristics can be quantitatively reflected, so as to achieve a qualitative leap in disease diagnosis.

Spectral CT consists of five primary tools: spectral curves, mono-energetic images at different keV levels, material decomposition and quantitative analysis, effective atomic number (Eff-Z) images, and virtual non-contrast (VNC) images. The first is spectral curves. The spectral curve is also known as the attenuation curve of CT value, which means that X-rays decay after passing through materials. Different keVs correspond to different CT values. Spectral CT (e.g., GE Revolution CT) can generate 141 different CT values between 40 and 140 keV, and these values are connected into a curve, which is the energy spectrum curve (Chen et al., 2019). Different tissues, organs, and lesions have their own unique spectral curves. The shape and slope of the energy spectrum curves can be used to judge the nature of the lesion and the homology of the tumor, it can provide more valuable information for the clinic. Secondly, the X-ray beam of a conventional CT scan produces a mixed-energetic image, but spectral CT can provide multiple sets of mono-energetic images at different keV levels. Currently, some studies have shown that mono-energetic images can effectively improve the signal-to-noise ratio (SNR) and contrast-noise ratio (CNR), especially in obese patients (Matsumoto et al., 2011; Atwi et al., 2019; Große Hokamp et al., 2019). Low keV mono-energetic images can increase the contrast between different tissues, which is conducive to the detection of small lesions with similar density to parenchymal organs (Parakh et al., 2018). But the high keV can effectively reduce metal hardening artifacts, especially when combined with multiple artifact reduction system (MARS) technologies, which can effectively remove metal implant artifacts, providing higher image quality for clinical diagnosis (Bamberg et al., 2011; Meinel et al., 2012; Laukamp et al., 2018; Kim et al., 2019). The third important spectral CT capability is material decomposition and quantitative analysis. Spectral CT can be based on different substances in X-ray absorption, the use of base substances (such as water, iodine, calcium, uric acid (UA), and blood, etc.) for material quantitative analysis, can be used to quantify the iodine concentration (IC) in a given region after enhanced scanning, so as to assess the blood supply of the lesion (McCollough et al., 2015; Lu et al., 2019). The fourth is Eff-Z. The Eff-Z analysis of spectral CT can analyze the components of inorganic substances, and obtain the effective atomic number of inorganic substances within the Region of interest (ROI), so as to make a qualitative diagnosis (McCollough et al., 2015). The last one is VNC images. VNC images are created by subtracting all contrast enhancement structures from the acquired image. So VNC images appear as an alternative to conventional true unenhanced phase images, which can effectively reduce the radiation dose delivered to patients (Jamali et al., 2019).

## GI cancers specific applications

### Esophageal cancer

Esophageal cancer (EC) is a relatively common malignant cancer of the digestive tract, the incidence rate is the seventh cancer and the sixth in mortality in the world (Sung et al., 2021). The pathological types were mainly divided into esophageal squamous cell carcinoma and esophageal adenocarcinoma. However, most patients with early EC miss the optimal treatment period due to the lack of typical clinical manifestations and are diagnosed in the advanced stages. Surgery is usually used for early esophageal cancer (T1 and T2), while neoadjuvant chemotherapy followed by surgery is recommended for advanced EC (T3 and T4) (Shapiro et al., 2015). Therefore, accurate preoperative staging is particularly important for determining the treatment of patients with EC (Rice et al., 2016; Guo et al., 2020). CT remains the primary noninvasive method for preoperative T staging in patients with EC, according to the American Joint Committee on Cancer (AJCC) guidelines (Edge and Compton, 2010). However, traditional CT cannot accurately display the boundary of the lesion, and the preoperative staging is very limited. The number of studies on EC with spectral CT has been growing, mainly on the T staging and efficacy evaluation of EC.

#### T staging

T staging is one of the popular research interests in the field of EC. Cheng et al. (2023) found that 40 keV mono-energetic images in the venous phase effectively improved tumor visualization and were significantly better than conventional mixed-energetic images in T staging. The agreement between 40 keV mono-energetic images at the venous phase and pathologic T categories was 81.63%, but the agreement between conventional mixed-energetic images and pathologic T categories was 48.97%. 40 keV mono-energetic images at the venous phase effectively improve the diagnostic efficiency to identify T1-2 from T3-4 in EC patients. In the staging of patients with EC, it is important to evaluate the depth of tumor invasion and the infiltration of extra-esophageal tissue. Zopfs et al. (2021) research found that for the evaluation of EC invasion depth, the Likert scores of mono-energetic images in 40–50 keV and iodine overlay images of spectral CT were significantly higher than those of conventional CT imaging, for tumor delineation, iodine overlays provided an optimal assessment. They found that the combination of all spectral data yielded a sensitivity, specificity, accuracy, positive and negative predictive value for detection of advanced tumor infiltration (T3/T4) of 42.4% (95 %-CI: 32.2%–53.1%), 82.0% (95 %-CI: 68.5%–91.4%), 56.3% (95 %-CI: 47.8%–64.6%), 81.3% (95 %-CI: 67.4–91.1) and 43.6 (95 %-CI: 33.4–54.2). Although not as accurate as ultrasound diagnosis of T staging.

#### Prognosis evaluation

Spectral CT iodine map performed before and after chemoradiotherapy (CRT) of EC can be used as a traditional morphological indicator to evaluate the prognosis (Ge et al., 2018). Ge et al. (2018) evaluated the effect of CRT on EC by IC of spectral CT and standard CT values. They found that after CRT treatment, the normalized concentration of iodine (NIC) in arterial and venous phases and normalized CT (NCT) values of the lesions in the effective group were lower than before treatment, and the NIC in venous phase and NCT values in the ineffective group were also lower than before treatment, confirming the esophageal the concentration of iodine can functionally assess the efficacy of CRT. The iodine map can directly reflect the difference in the concentration of iodine in the tumor and indirectly reflect the blood supply in the lesion. Quantitative analysis with the concentration of iodine not only can improve the diagnostic accuracy, but also specify the target lesions in patients with EC (Gao et al., 2016).

### Gastric cancer

Gastric cancer (GC) is the fifth most common cancer and the third leading cause of cancer-related deaths worldwide (Sung et al., 2021). About 95% of GCs are gastric adenocarcinoma (Dicken et al., 2005). In China, the incidence and mortality rates associated with gastric adenocarcinoma are both the highest among all malignant tumors of the digestive tract (Wong et al., 2004). Most of the patients with GC are initially diagnosed as an advanced disease and only 25% are resectable at presentation, with limited 5-year survival to 20% (Song et al., 2017). Therefore, early diagnosis and treatment are particularly important for patients with GC. In clinical practice, the gold standard to obtain the diagnosis of GC is still through preoperative endoscopic biopsy, but endoscopic biopsy is an invasive examination, and there is inevitable sampling bias in the process of endoscopic biopsy (Liu et al., 2017). So, CT with multiplanar reconstruction still is currently the most common and effective method to stage GC, including assessing locoregional tumor invasion and discovering adjacent and distant metastases (Filippone et al., 2004; Edge and Compton, 2010; Makino et al., 2011). The application of spectral CT in GC has been a promising area for research with a rising number of relevant studies published every year.

#### Diagnosis

**FIGURE 2:**
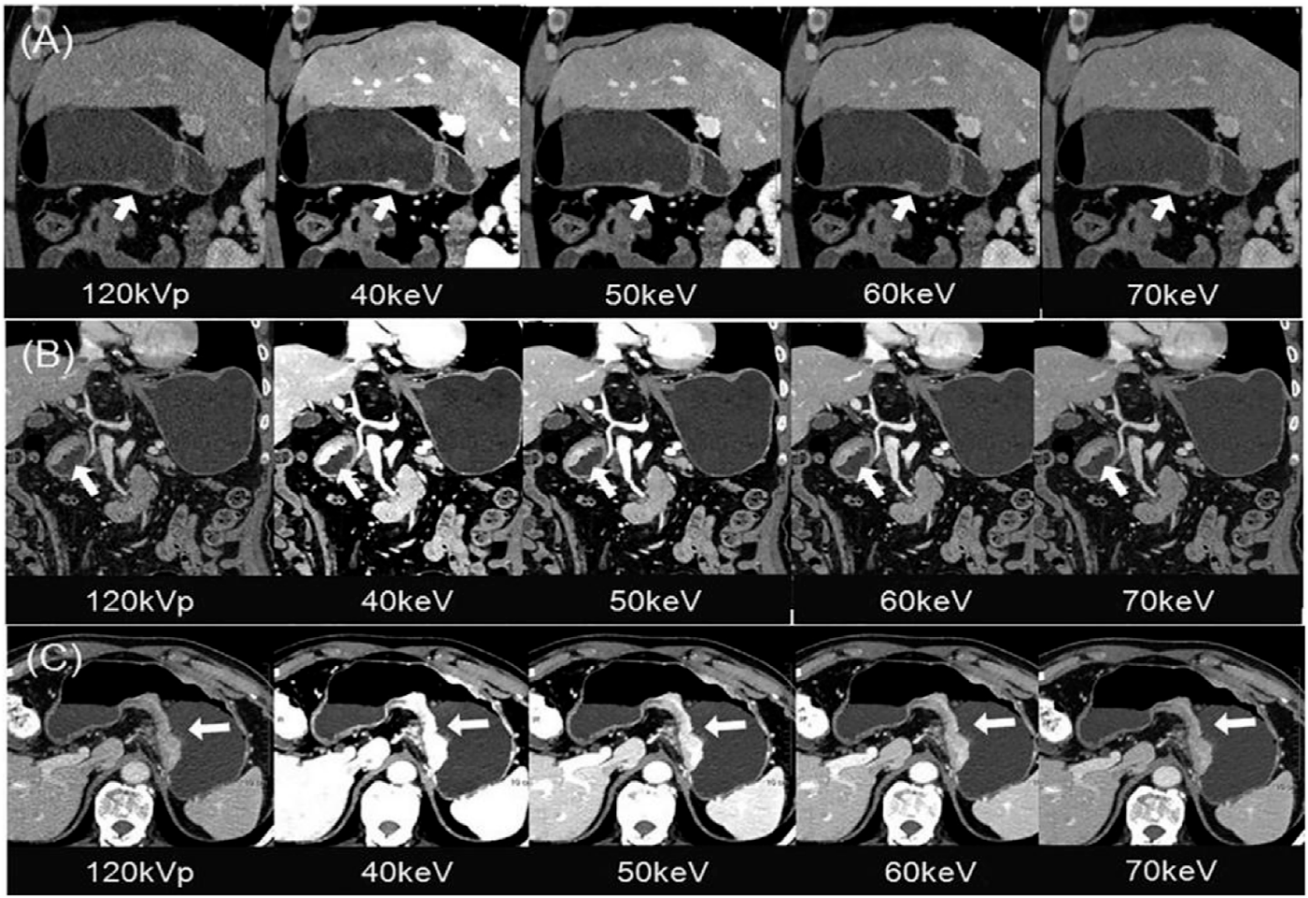
Gastric cancer patients with 120 kVp polyenergetic images and 40–70 keV VMIs. **(A)** Sagittal MPR images showed a small superficial elevated lesion at the antrum of the stomach, which was approved as T1 by pathology. The lesion was visualized better from 40–60 keV for increased lesion attenuation, compared with traditional 120 kVp images. **(B)** Coronal MPR images showed local gastric wall thickening with hyperenhancement at the antrum of the stomach, which was approved as T2 by pathology. The infiltration depth was determined more easily and confidentially in the 40–60 keV VMIs, of which the enhanced lesion was evidently illustrated to be confined in gastric wall without abutting on the outer contour. **(C)** Axial images showed diffuse gastric wall thickening with hyper-enhancement at the posterior wall of the stomach, which was approved as T4a by pathology. The perigastric fat infiltration is visualized more clearly in the 40–60 keV VMIs compared with the traditional 120 kVp images. Of all three images, 40 keV VMIs performed the best. Abbreviations: VMI, virtual monoenergetic images; PEI, polyenergetic images; MPR, multiplanar reconstruction (Liu et al., 2020).

An important preoperative topic of GC is the diagnosis, mainly the histological types of GC, benign and malignant differentiation, and preoperative T staging. Li et al. (2018a) found through a retrospective study that the values of IC at the arterial phase (ICAP), IC at the portal venous phase (ICPP), normalized IC at the arterial phase (NICAP), and normalized IC at the portal venous phase (NICPP) were significantly higher in the poorly-differentiated group than those in the well-differentiated group. And the AUC values of ICAP, NICAP, ICPP, and NICPP in discriminating poorly and well-differentiated gastric adenocarcinoma were 0.756, 0.919, 0.851, and 0.684, respectively, especially NICAP demonstrated the greatest ability in discriminating the histological types of gastric adenocarcinoma. For differentiating between benign and malignant GC, the values of the ICAP, NICAP, ICPP, and NICPP in the GC group were significantly higher than those in the benign gastric wall lesions group. Of note, NICPP demonstrated the greatest ability in discriminating GC, and the optimal cut-off values of NICPP was 0.364, the sensitivities and the specificities were 91.95% and 87.96%, respectively. By comparing the accuracy of spectral CT mono-energetic images and conventional mixed-energetic images for the T stages of GC, Xie et al. (2018) found that the diagnostic accuracy of mono-energetic images was higher than that of conventional mixed-energetic images, especially for the T3 and T4 stages of GC. Liu et al. (2020) also revealed that the 40 keV mono-energetic images were better for identifying the invasion depth in advanced GC (T2-4), for which the T staging accuracy was increased by 28.6% (Figure 2).

#### Lymph node status

Accurate prediction of the lymph node (LN) status of GC, which is a remarkable prognostic factor, is important to determine the appropriate treatment. Xie et al. (2018) found that for LN staging, the general accuracy of the mono-energetic group and the conventional mixed-energetic group was 70.4% and 64.8%, respectively. Li et al. (2018b) developed and internally validated a spectral CT-based nomogram to predict LN metastasis in patients with GC. They found that the nomogram was significantly associated with LN status, and the tumor thickness, Borrmann classification, and the IC at the venous phase were independent predictors of LN metastasis, which can be used to facilitate the preoperative individualized prediction of LN metastasis in patients with GC. Li et al. (2020) built a spectral CT imaging-based radiomics nomogram by deep learning method for LN metastasis prediction in GC. According to the experimental results, the spectral CT radiomics signature was associated with LN metastasis in the arterial phase (AP) and venous phase (VP), and an achieved area under the ROC curve (AUC) of 0.71 for AP and 0.76 for VP in the test set.

#### Treatment response

Many studies focused on the prediction of the treatment response of patients. Wang et al. (2021) developed and validated an iodine map radiomics model, which was superior to the radiomics model built by conventional mixed-energetic images, the AUC was 0.940 and 0.953, respectively. Indicating the discrimination value of iodine from spectral CT for serosal invasion in GC patients after neoadjuvant chemotherapy. Liu et al. (2021) build and assess a pre-treatment spectral CT-based clinical-radiomics nomogram for the individualized prediction of clinical response to systemic chemotherapy in advanced GC. They found that clinical stage and IC value were independent clinical predictors of response to chemotherapy for advanced GC. The multi-energy radiomics model predicting response probability was superior to two monochromatic radiomics models and the clinical model, the AUCs of them were 0.934, 0.914, and 0.774, respectively. So radiomics combined whit spectral CT may serve as a promising technique for predicting the response to treatment in patients with advanced GC.

Other related factors of GC have also been investigated in previous studies. The expression of the Ki-67 antigen is considered to be associated with clinicopathological characteristics of gastric adenocarcinoma. Cheng et al. (2018) found that IC, NIC, and the curve slope values were found significantly different among the low-, medium- and high-level Ki-67 expression groups in VP and delayed phase (DP), and the higher grades of Ki-67, the bigger values of these parameters. For further analysis, they found the correlation between Ki-67 grade and IC, NIC, and the curve slope values. Zhao et al. (2021) developed and evaluated a spectral-based nomogram for noninvasive identification of the status of human epidermal growth factor receptor 2 (HER2) expression in GC. They found that the IC at the venous phase (ICVP) and the normalized IC at the venous phase (NICVP) were significantly lower in the group with HER2-positive GC than in the group with HER2-negative GC. The newly built nomogram was a promising tool for noninvasive stratification of HER2 status, which could provide strong clinical evidence for HER2-directed therapy and personalized patient management in a short period of time.

### Colorectal cancer

Colorectal cancer (CRC) is the third most common kind of cancer and the second leading cause of cancer-related fatalities worldwide (Sung et al., 2021). It is crucial to carry out research on the diagnosis, treatment response prediction, and survival prediction of CRC, which can improve the prognosis of patients and significantly reduce the social and medical burden. In recent years, promising research results have emerged in the preoperative, intraoperative, and postoperative stages of CRC using spectral CT, covering the entire process of CRC diagnosis and treatment.

#### Diagnosis

Spectral CT has unique advantages in the diagnosis of CRC. Extramural vascular invasion (EMVI), one of the most important therapy evaluation goals, is related to tumor microcirculation. Gao et al. (2023) held that iodine quantification using spectral CT, especially the NIC to the aorta of the tumor, differs between the EMVI-positive and EMVI-negative groups and seems to help predict the EMVI of rectal cancer. Kang et al. (2018) also found quantitative IC from spectral CT are significantly correlated with PCT parameters, with better Intra- and interobserver agreements and lower radiation doses, indicating that the clinical applicability of spectral CT might be expanded to assess the hemodynamic status of CRC.

Microsatellite instability (MSI) function is a predictive biomarker for clinical outcomes and predicts responses to adjuvant 5-fluorouracil and immunotherapy in CRC. Multiple parameters in spectral CT imaging combined by Wu et al. (2019a) provides relatively high diagnostic accuracy for discriminating MSI, with AUC of 0.886. A radiomics model based on iodine-based material decomposition images derived from spectral CT imaging was also created by them, achieved a high AUC (training: 0.961; validation: 0.918; testing: 0.875), which was deemed to aid in locating individuals who might benefit from chemotherapy or immunotherapy (Wu et al., 2019b).

Based on multi-keV of spectral CT, virtual monoenergetic imaging (VMI) is usually used to improve image quality. Lenga et al. (2020) demonstrated that 40 keV VMI improves the reliability (ICC: 0.88) and diagnostic accuracy (89.1%) for the detection of colorectal liver metastases (CRLM), and their further study concluded that 40 keV VMI combined with noise optimization could better display lesion contours in CRLM (Lenga et al., 2018).

#### Efficacy evaluation

Spectral CT is also applied for early evaluation of chemotherapy efficacy in patients with CRLM. Although MRI and PET/CT have good performance in the early response evaluation of chemotherapy for CRLM, the high cost of them precludes their use in most patients with CRLM, because of multiple imaging examinations during chemotherapy. Li et al. (2023) showed that IC of spectral CT, as independent risk factor for overall survival (OS) in CRLM patients (hazard ratio [HR]: 1.238), could well predict the early response of first-line chemotherapy combined to CRLM, using IC cutoff values of 4.75 (100ug/cm3) (AUC: 0.916), which meant that spectral CT could replace MRI and PET/CT to monitor the chemotherapy efficacy of CRLM in early stage.

#### Lymph node status

**FIGURE 3:**
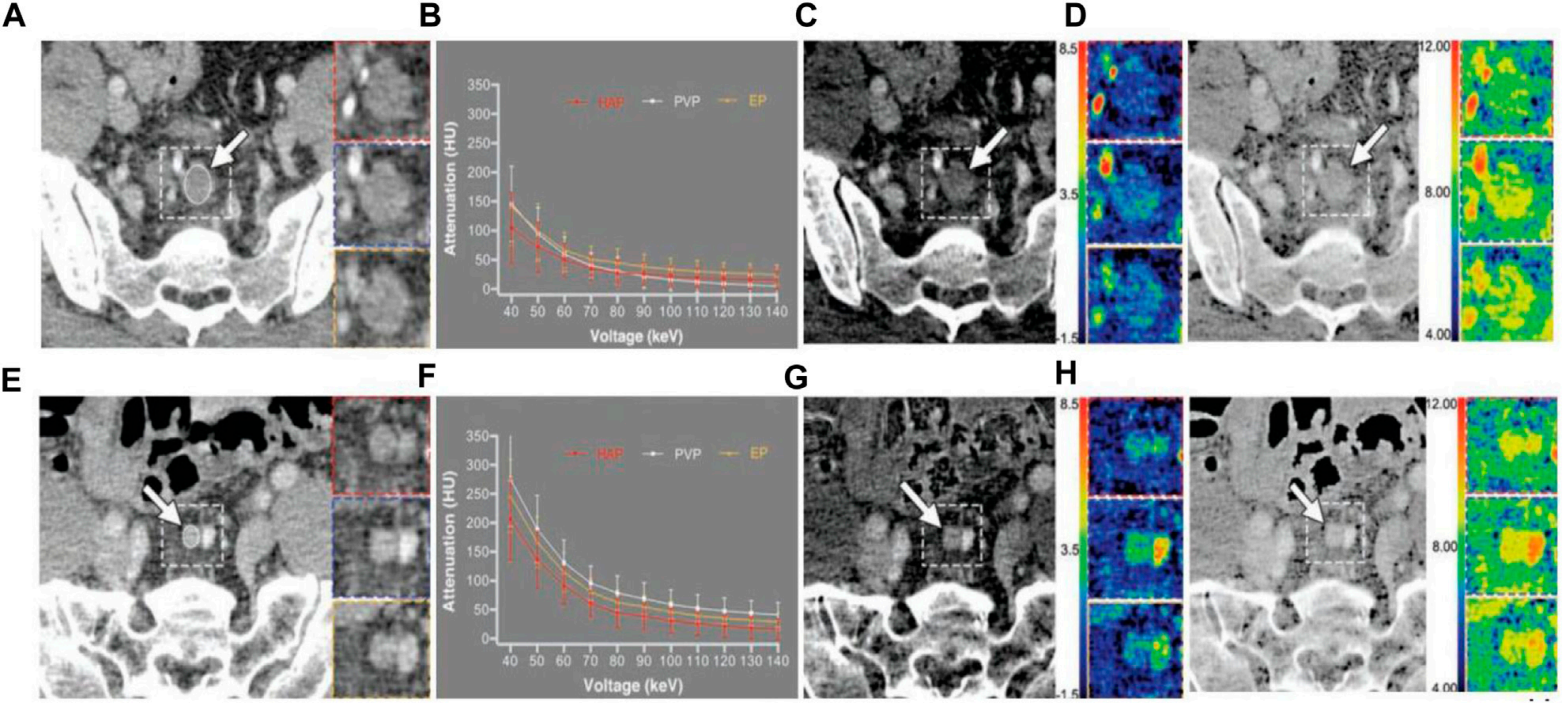
42-year-old man with poorly differentiated pathologic T3N2 rectal adenocarcinoma. **(A–D)** is a representative dual-energy CT examination of metastatic lymph node. Dashed line indicates location of inserts. Inserts show HAP (red), PVP (blue), and EP (yellow) images. **(A)**, PVP 70 keV monochromatic contrast-enhanced CT images show largest metastatic lymph node (arrow) around superior rectal artery with short-axis diameter of 12.1 mm. ROI (oval) was drawn to cover entire lymph node; area is 59.2 mm2. **(B)**, Graph shows spectral attenuation curves of metastatic lymph node. Slopes of attenuation curves are 1.70 in HAP, 3.36 in PVP, and 3.15 in EP. **(C)**, PVP contrast-enhanced iodine-based material decomposition CT images show iodine concentrations are 0.81 ± 0.72 mg/cm3 in HAP, 1.70 ± 0.67 mg/cm3 in PVP, and 1.58 ± 0.75 mg/cm3 in EP. Arrow indicates largest metastatic lymph node. **(D)**, PVP contrast-enhanced Eff-Z CT images show Eff-Zs are 8.08 ± 0.45 in HAP, 8.61 ± 0.36 in PVP, and 8.54 ± 0.43 in EP. Arrow indicates largest metastatic lymph node. 63-year-old man with moderately differentiated pathologic T2N0 rectal adenocarcinoma. **(E–H)** is a representative dual-energy CT examination of nonmetastatic lymph node. Dashed line indicates location of inserts. Inserts show HAP (red), PVP (blue), and EP (yellow) images. **(E)**, PVP 70 keV monochromatic contrast-enhanced CT images show largest, metastatic lymph node (arrow) around superior rectal artery with short-axis diameter of 6.7 mm. ROI (oval) was drawn to cover entire lymph node; area is 23.6 mm2. **(F)**, Graph shows spectral attenuation curves of nonmetastatic lymph node. Slopes of attenuation curves are 4.86 in HAP, 5.97 in PVP, and 5.53 in EP. **(G)**, PVP contrast-enhanced iodine-based material decomposition CT image shows iodine concentrations are 2.45 ± 0.77 mg/cm3 in HAP, 3.02 ± 0.81 mg/cm3 in PVP, and 2.81 ± 0.67 mg/cm3 in EP. Arrow indicates largest metastatic lymph node. **(H)**, PVP contrast-enhanced Eff-Z CT images show Eff-Zs are 9.02 ± 0.37 in HAP, 9.26 ± 0.36 in PVP, and 9.18 ± 0.30 in EP. Arrow indicates largest metastatic lymph node. Abbreviations: HAP, hepatic arterial phase; PVP, portal venous phase; EP, equilibrium phase; Eff-Z, effective atomic number (Yang et al., 2019).

LN metastasis, which is a key prognostic factor for CRC, is among the other study topics of CRC. Sato et al. (2019) calculated NIC in the largest pararectal LNs (PRLN) and lateral pelvic LNs (LPLN) in patients with rectal cancer and demonstrated that NIC reduction is helpful in predicting positive metastasis of PRLN and LPLN. In studies of preoperative diagnosis of regional LNs in CRC by combining NIC and Eff-Z, Yang et al. (2019) found that the combination of the two can further improve the accuracy of predicting LN metastasis (Figure 3), while Qiu et al. (Yang et al., 2021) believes that Eff-Z is not significantly helpful in judging metastatic and non-metastatic LNs. Wang et al. (2022a) used the radiomics to conduct feature selection and diagnostic analysis of spectral CT images and obtained that the feature based on 120 kVp-like images (AUC: 0.922) and iodine (water) image (AUC: 0.866) had good diagnostic performance in predicting CRC LN metastasis. By combining spectral CT parameters and clinical factors, Cao et al. (2021) developed a spectral CT nomogram with an AUC of 0.876 in the training cohort and 0.852 in the validation cohort. It shows that this column diagram has good efficacy in clinical applications. All of these studies have shown that spectral CT has good performance in the prediction of LN metastasis.

### Liver cancer

Primary liver cancer is the sixth most prevalent form of cancer and the third most lethal cancer globally, and the first and second major pathological types are hepatocellular carcinoma (HCC) and intrahepatic cholangiocarcinoma (ICC) (Sung et al., 2021). The incipient symptoms of primary liver cancer are usually a typical, so many patients are at Barcelona clinic liver cancer stage-C at diagnosis and have lost the opportunity for curative surgery (Park et al., 2015; Bruix et al., 2016). Early diagnosis, individual assessment, and prognosis prediction are very important in clinical practice. With the gradual maturity of spectral CT, research on the diagnosis and treatment of liver tumors has emerged one after another.

#### Diagnosis

Spectral CT can effectively identify the pathological types of liver tumors before surgery. Mahmoudi et al. (2022) found through a retrospective study that the ICAP, normalized iodine uptake (NIU), and a fat fraction (FF) between HCC and ICC had significant differences. The ICAP to differentiate HCC and ICC revealed the highest ability, the AUC was 0.93, and NIU followed (AUC: 0.87). For ICAP, the optimal threshold of 2.33 mg/dL to discriminate between HCC and ICC with a sensitivity of 89.36% and specificity of 76.6%. Kaltenbach et al. (2018) also found the NIU of the HCC was higher than that of the hepatic neuroendocrine tumors (NET) metastases. The optimal threshold of NIU was 0.22, the sensitivity was 100%, and the specificity was 90%.

In recent years, the combination of deep learning and spectral CT has been used more and more in the diagnosis of liver cancer. Bae et al. (2023) through combined low monoenergetic images and deep learning denoising, could obtain high conspicuity of HCC and acceptable image noise while lowering the amount of contrast medium. They thought that the findings on noninferiority for the 50 keV deep learning denoising images in terms of image quality and lesion conspicuity, compared with model-based iterative reconstruction images of the standard-contrast-dose group, may contribute to the wider clinical use of low monoenergetic images for contrast dose reduction. Narita et al. (2023) evaluated the clinical application of HCC by iodine map generated from deep learning-based spectral CT imaging, and they found that it could effectively improve the contrast-to-noise ratio of hepatic arterial stage images and effectively evaluate the vascular distribution of HCC in an arterial stage. Wang et al. (2022b) designed a new deep learning model called MVI-Mind, which consists of a lightweight transformer for segmentation and a convolutional neural network for predicting microvascular invasion, and used several deep learning techniques to compare the proposed methods. The AUC values of MVI-Mind in arterial phase, portal vein phase, and delayed phase CT images were 0.9223, 0.8962, and 0.9100, respectively, which significantly improved the prediction accuracy compared with other deep learning segmentation algorithms.

#### Efficacy evaluation

Accurate evaluation of the patient’s treatment effect is essential to achieve individualized treatment for different stages of HCC. Yue et al. (2021) found that NICAP and NICPP have high sensitivity and specificity in distinguishing tumor active area (TAA), adjacent normal hepatic parenchyma (ANHP), and tumor necrotic area (TNA), and could reflect the perfusion information of liver tissue. The quantitative parameters of spectral CT may help physicians to better judge the effect of treatment and provide quantitative information as a supplement to mRECIST and LI-RADS, to make correct clinical decisions. Choi et al. (2021) quantified iodized oil retention in tumors after transarterial chemoembolization using spectral CT imaging in patients with HCC, and evaluate its performance in predicting 12-month tumor responses. They found that the presence of suspected residual tumors was the only significant factor associated with a non-complete response, with an odds ratio of 72.0.

### Pancreatic cancer

Pancreatic cancer, with its low incidence but high mortality rate, is the seventh most deadly cancer worldwide (Sung et al., 2021). Surgical resection constitutes the only means that can lead to prolongation of life (Witkowski et al., 2013). However, most patients have already lost the opportunity for surgery because they are already at an advanced stage or have metastases. Therefore, the early diagnosis of the disease is very important and urgent. In recent years, with the wide application of spectral CT, the diagnosis and survival prediction of pancreatic cancer have been discussed.

#### Diagnosis

Pancreatic ductal adenocarcinoma (PDAC) is the most common pathological type of pancreatic cancer, resulting in a high mortality rate (Sung et al., 2021). Achieving an accurate diagnosis of PDAC contributes significantly to avoiding false predictions and improving patient survival outcomes. For distinguishing between PDAC and benign pancreatic lesions (e.g., pancreatic simple cyst, cystadenoma and intraductal papillary mucinous neoplasm (IPMN)) using spectral CT scans, Ebrahimian et al. (2022) found that when IPMN and mucinous neoplasms were grouped with the malignant lesions, both the spectral CT radiomics and quantitative features had high AUCs (0.92 and 0.85, respectively). For distinguishing between PDAC and chronic pancreatitis, Yin et al. (2015) retrospective study showed a significantly higher iodine concentration in chronic mass-forming pancreatitis compared with PDAC on both arterial and pancreatic phase images (mean NIC, 0.07 ± 0.02 mg/mL vs. 0.28 ± 0.04 mg/mL; 0.26 ± 0.04 mg/mL vs. 0.53 ± 0.02 mg/mL, respectively).

Preoperative accurate localization of PDAC, tumor scope, and accurate identification of blood supply arteries are also important. Nagayama et al. (2020) study demonstrated that the mono-energetic images quality of spectral CT is obviously better than that of traditional mixed-energetic images, the 40 keV mono-energetic images provides the best quality for PDAC evaluation because it has high pancreatic tumor contrast and vascular opacity without the associated increase in image noise. Low keV mono-energetic images are significantly superior to traditional mixed-energetic images in tumor conspicuity, margin delineation and visualization of peripheral blood vessels, and have great advantages for early diagnosis and local staging of PDAC. Liang et al. (2022) also found that low keV on spectral CT effectively improved the visualization of pancreatic supplying arteries, and a clearer display of the anatomy and variation of these arteries was essential for intraoperative guidance.

#### Lymph node status

LN metastasis also has a high prognostic value in pancreatic cancer, especially the early accurate prediction of its status is particularly important. An et al. (2022) developed a deep learning model that extracted different radiomic features from spectral CT scans to predict LN metastasis using a pre-trained ResNet-18 model. Compare the effects of different approaches by adding key clinical features to the experiment. The combined model combining deep learning features and key clinical features had the highest AUC at 0.92 and an accuracy of 86%.

#### Treatment response

**FIGURE 4:**
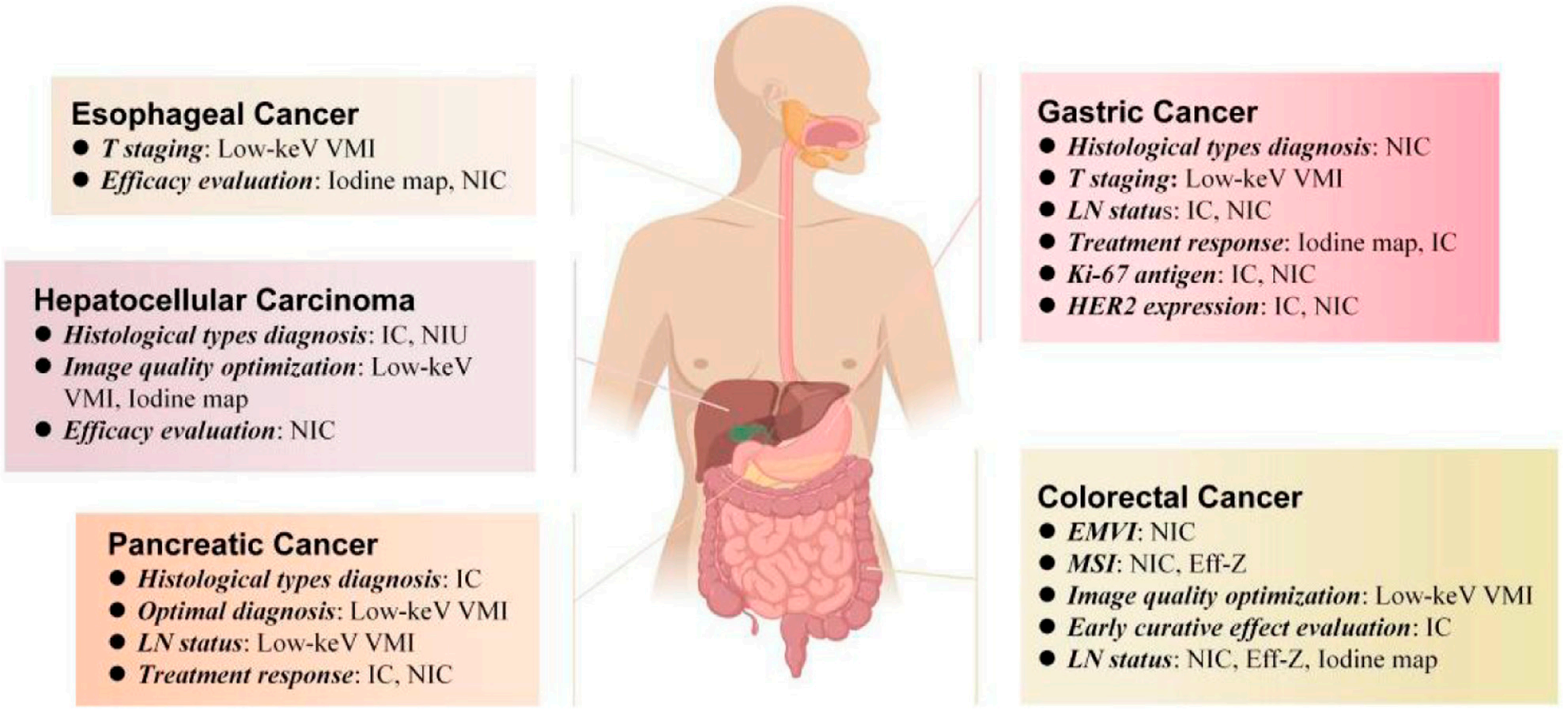
Spectral CT quantitative parameters for preoperative diagnosis, differentiation, LN status and treatment response of different types of gastrointestinal tumors, including low-keV virtual monoenergetic imagies (low-keV VMI), iodine map, iodine concentration (IC), normalized concentration of iodine, effective atomic number (Eff-Z), normalized iodine uptake (NIU).

Treatment response is also important for the prognosis of pancreatic cancer. Noda et al. (2018) compared the maximum diameter, CT number, IC, mean CT number (△HU), and mean IC (△IC) of PDACs between those who response and non-response group to chemotherapy. They found that the maximum diameter, CT number and IC of the response group were smaller than those of the non-response group, but the △HU and △IC of the responsive group were significantly higher than those of the non-responsive group, and the AUC and sensitivity of all the diagnostic factors of IC were the highest (0.889, 97.7%, respectively). Fukukura et al. (2020) though obtained iodine density images from equilibrium-phase spectral CT for derived extracellular volume (ECV) fractions. It was an independent predictor of progression-free survival and overall survival in patients with stage 4 PDAC treated with chemotherapy on multivariate analysis. The spectral CT quantitative parameters involved in preoperative diagnosis, differentiation, LN status, and treatment response of all types of gastrointestinal cancer are summarized in Figure 4.

## Limitations of the application of spectral CT in GI cancers

Although spectral CT is widely used in gastrointestinal tumors, it still has some limitations. This article will elaborate on the limitations of the current application of spectral CT in gastrointestinal tumors from the characteristics of the gastrointestinal structure, scanning equipment, and application of deep learning and radiomics.

### Gastrointestinal structure

Firstly, the gastrointestinal tract is a hollow structure comprising of mucosa, submucosa, muscular layer, and outer membrane. In contrast to solid organs like the liver and kidney, its carcinogenesis primarily originates from the innermost mucosa and submucosa layers. When lesions are very small or in early stages, tumors tend to be smooth with minimal density variation compared to surrounding normal tissue. Consequently, spectral CT is still exhibit lower diagnostic sensitivity than endoscopy in such cases. Therefore, diagnosis of gastrointestinal tumors continues to rely on endoscopy as well as pathological puncture or resection. Secondly, it has been discovered that the degree of cancer cell invasion in gastrointestinal tumors is not solely determined by the display effect of imaging technology but also closely associated with the extent of lumen filling. Contraction of the lumen wall results in incomplete lumen filling, rendering layer identification challenging, while dilation of the lumen wall leads to excessive thinning, making early tumor invasion depth estimation impossible. Therefore, achieving optimal dilation of the gastrointestinal tract’s lumen wall and maximizing the potential role of spectral CT are crucial tasks in gastrointestinal CT examinations. Additionally, inhibiting peristalsis poses a significant challenge during gastrointestinal CT examinations. Peristalsis-inhibiting drugs can only partially slow down or inhibit gastrointestinal tract activity without completely halting its movement. This slight peristaltic activity may introduce certain interference when examining fine structures or performing dynamic assessments.

### Scanning instrument

Firstly, although CT scanning is highly valuable in medical diagnosis and treatment, the utilization of X-ray radiation may potentially induce adverse effects on the human body. Spectral CT presents a reduced radiation dose compared to conventional spiral CT; however, clinicians must engage in long-term repetitive follow-up observations to comprehensively investigate tumor development and progression. Additionally, multiple post-treatment evaluations are necessary for tumor patients to assess efficacy. The frequent implementation of CT scans escalates patient radiation risks, thereby limiting the applicability of spectral CT in longitudinal studies or follow-up examinations. Secondly, spectral CT essentially belongs to dual-energy CT and is implemented based on dual-energy technology. Using different dual-energy technologies, various manufacturers have developed different dual-energy CT systems (such as single-source dual-beam energy CT, dual-source dual-energy CT, dual-layer spectral detector CT, single-source instantaneous tube voltage switching dual-energy CT, photon counting CT). In addition to different CT systems, scanner, kVp settings, and other parameter settings may vary depending on the manufacturer and system. It may yet be explored whether the same results can be obtained when different studies use DECT systems with different Settings. Thirdly, although the resolution of spectral CT is greatly improved compared with that of traditional CT, it still has certain limitations in tumor edge identification. In related spectral CT studies, the judgment of tumor ROI is still based on the subjective intuition of the delineator. Although these contours are achieved by knowledgeable and experienced radiologists, this non-objective judgment also causes a certain bias to the research results. Finally, different spectral CT studies may involve different scanning protocols, such as virtual non-contrast scan instead of conventional non-contrast scan, intravenous injection of asodamine to obtain optimal gastric distension, and ventilation of the esophagus to achieve full filling of the esophageal wall, as well as different contrast agent injection protocols and different brands of contrast agent, which may cause differences in the results of these studies.

### Deep learning and radiomics

Firstly, deep learning requires a large amount of annotated data for training, however, the acquisition and annotation of spectral CT images is a complex and time-consuming task. Since spectral CT technology is relatively new, the available annotated data is relatively limited, which limits the application of deep learning algorithms. Secondly, the samples of gastrointestinal tumors are diverse, and the number of different types of tumors may be unbalanced. This will lead to poor learning effect of deep learning algorithms on samples of a few categories during training. Moreover, the selection and extraction of features may be affected by factors such as tumor location, shape, and size, thus affecting the accuracy of their diagnosis in gastrointestinal tumors. Thirdly, although deep learning algorithms perform well on training data, generalization performance on new, unseen data remains problematic. This means that the algorithm may perform differently on different datasets of gastrointestinal tumors or on different machines, requiring further validation and optimization. Finally, the black-box nature of deep learning algorithms limits the interpretability and interpretability of their results. This can be a challenge for doctors and patients, as they need to understand how the algorithms make diagnostic decisions.

### Others

Firstly, in most studies, tumor ROI delineation was performed based on CT images of a single layer rather than the entire tumor volume, which ignores the potential spatial heterogeneity of the tumor. In addition, the ROI based on a certain level of the tumor cannot match the pathological specimen completely, which causes some deviations in the research results to a certain extent. Secondly, most of the studies were retrospective and had small sample sizes. Therefore, retrospective studies may have sample selection bias and cannot truly reflect the distribution of clinical cases. And too small a sample size may exaggerate the consequences of the association, with some studies including no more than 20 patients, so the statistical significance is questionable. All these affect the accuracy of the experimental results. Thirdly, most of the articles we collected focused on relatively single pathological types of gastrointestinal tumors, and most of them focused on common pathological types, such as esophageal squamous cell carcinoma, gastric adenocarcinoma, HCC, PDAC, colorectal adenocarcinoma, etc., without considering patients with other histological types of gastrointestinal tumors and case types with different degrees of differentiation. Last but not least, almost all of the studies are single-center studies, and the acquisition parameters of spectral CT images in each research center are quite inconsistent, which leads to the question of repeatability. Most of the findings require large multicenter independent cohort studies to verify the accuracy of the experimental results.

## Application prospect of spectral CT in GI cancer

The development of spectral CT is considered a milestone in CT diagnosis, multi-parameter imaging of spectral CT provides rich imaging data for the diagnosis of GI cancer. Among them, mono-energy imaging and substrate imaging (the most widely used is IC) have certain effects on improving the detection rate of early GI cancer, differential diagnosis of advanced GI cancer, evaluating the type, grade, and stage of GI cancer, evaluating the chemotherapy effect of GI cancer, and reducing the dosage of contrast agents and radiation dose of patients, greatly improving the detection rate and qualitative accuracy of lesions. The following is the future application prospect of spectral CT in gastrointestinal diseases.

### Photon-counting CT

Photon-counting CT (PCCT) uses a photon-counting detector (PCD) to detect the energy of photons in X-rays and count the number of them, and then dissociate different single-energy images. Unlike conventional CT, which uses scintillation crystals as detector materials, PCCT detectors use semiconductor materials such as cadmium telluride. This semiconductor material can directly convert X-rays into photons while distinguishing the energy of the X-rays and counting the number of photons (Kreisler, 2022). PCCT is the most advanced version of CT known.

Traditional spectral CT can realize simultaneous identification and quantitative analysis of up to two substances based on dual-energy imaging. The analog calculation can obtain iodine maps, calcium maps, VNC imaging, virtual mono-energy image, and so on for clinical diagnosis. By setting multiple thresholds (T0, T1, T2, T3), PCCT can simultaneously read CT data in different energy domains. This multi-energy imaging method provides the possibility for specific material imaging and multi-material decomposition (Mauro et al., 2021). For example, the simultaneous separation of two different contrast agents: iodine and gadolinium, iodine and bismuth, or other heavy elements (tungsten and nano-gold, etc.) enables simultaneous imaging of multiple contrast agents and targeted molecular imaging. In addition, the CNR of images can be further improved by optimizing the weighting of different energy domains, especially in enhanced CT. In addition, it can record spectral information, provide color images and multi-information images, and the image resolution is higher, but also can greatly reduce the radiation dose, and the noise is lower when working.

The higher resolution and more accurate material separation of PCCT can better provide the detection rate of gastrointestinal tumors, improve the accuracy of tumor localization, qualitative and staging diagnosis, and better evaluate the tumor efficacy. However, how it can provide a more advantageous diagnosis for early, small, and hidden gastrointestinal cancers needs more research to further explore. Lower radiation dose imaging with PCCT is important for long-term follow-up and screening of intestinal tumors, which can reduce radiation dose and risk to patients.

### Molecular image probe

The molecular imaging probe plays a pivotal role in molecular imaging using CT, representing the most crucial component. Typically, CT molecular imaging probes consist of materials with high X-ray absorption capacity, enabling imaging through the measurement of X-ray absorption and scattering. Although iodine is commonly employed as a CT probe, its toxicity to the human body poses concerns. Low molecular weight iodine is rapidly eliminated by the kidneys, resulting in very short contrast times; moreover, X-rays catalyze ionization of iodine ions, potentially causing additional harm to humans. The integration of novel nanomaterials and biotechnology can address these limitations and enhance the capabilities of CT probes.

**FIGURE 5:**
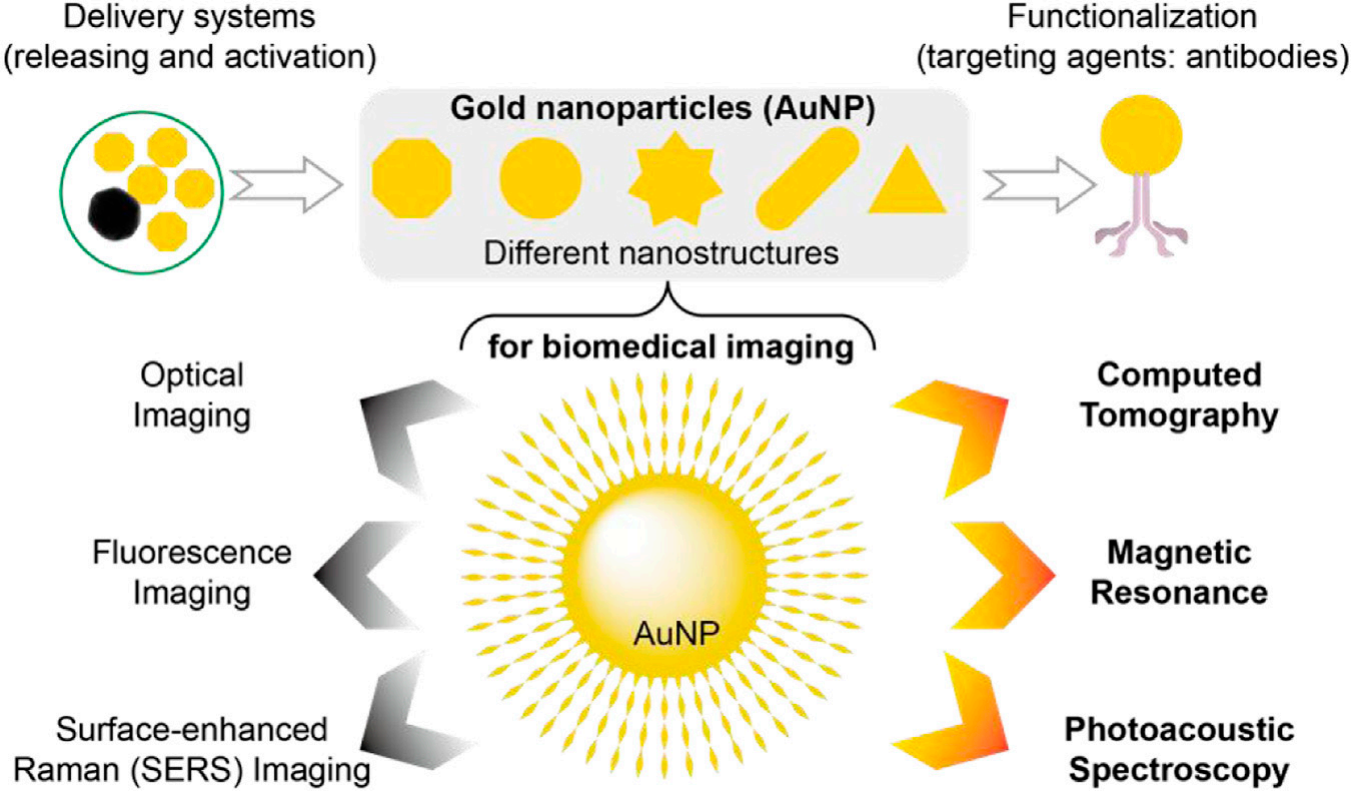
AuNP have been widely developed as contrast agents, therapeutic diagnostic platforms, and molecular imaging probes. This popularity has resulted in a large number of AuNP designs with different sizes, shapes, surface functionalization, and assembly, including novel structural AuNP, targeted AuNP for cancer imaging, and other AuNP, all to very closely match the requirements of various imaging applications. Thus, AuNP-based molecular imaging probes allow the use of CT, fluorescence, optical, and surface-enhanced raman imaging, photoacoustic spectroscopy, and MRI, as well as other novel technologies.

Gold nanoparticles (AuNP) are extensively utilized due to their excellent biocompatibility and physicochemical properties. Notably, their chemical inertness, photoelectric characteristics (such as Raman scattering, fluorescence, and surface plasmon resonance (SPR)), modifiability of the surface, and strong interaction with mercapto compounds offer significant potential in tumor diagnosis and therapeutic applications (Figure 5) (Yeh et al., 2017). Besides gold, other metallic elements such as 56Ba, 73Ta, 74W, 83Bi, 64Gd, and 70Yb (Wang et al., 2019) can also serve as CT probes for CT imaging by modifying them owing to their high X absorption coefficients. Furthermore, emerging molecular probes like germanium compounds (Nampi et al., 2021; Wang et al., 2023), yttrium compounds (Bychkova et al., 2022), ferrite nanoparticles (Litjens et al., 2017), oxygen sensors, etc., have been explored for CT imaging. Additionally, different types of molecular probes can be combined to achieve multimodal imaging using multimodal probes; for instance fluorescence-spectral CT dual-mode probes enable simultaneous fluorescence imaging and spectral CT imaging while nuclide-spectral CT dual-mode probes allow both nuclide imaging and spectral CT imaging.

The combination of molecular probes and spectral CT holds promising prospects for the application in gastrointestinal tumors. Molecular probes can effectively label specific molecular markers expressed by gastrointestinal tumor cells, such as HER2 in gastric cancer. By combining spectral CT with molecular imaging probes, targeted identification and localization of tumors can be achieved. Moreover, when combined with molecular probes, spectral CT enables the labeling of specific molecular markers in tumor blood vessels, facilitating a comprehensive evaluation of tumor blood supply and angiogenesis to provide a more accurate basis for treatment selection. Additionally, molecular probes can label specific metabolites within tumor cells which, when combined with spectral CT, further allows for an assessment of metabolic activity in tumors to monitor and evaluate therapeutic response and efficacy.

### Artificial intelligence

In recent years, artificial intelligence (AI) has emerged as a prominent research area in computer-aided diagnosis, wherein medical image data is processed and analyzed using advanced AI techniques to extract valuable information that aids doctors in making accurate diagnoses and treatment decisions. Among various AI technologies, deep learning (DLR) based on convolutional neural network (CNN) has gained significant popularity for medical image analysis due to its ability to accomplish tasks such as image classification, object detection, segmentation, and reconstruction. By training deep learning models with extensive medical image datasets, the inherent features and patterns within images can be automatically learned, enabling precise identification and analysis of diseases and abnormalities. Additionally, generative adversarial network (GAN), reinforcement learning (RL), multimodal fusion techniques, along with natural language processing (NLP), are also pivotal for the application of artificial intelligence in the field of medical imaging.

In spectral CT, artificial intelligence technology is primarily utilized in two key areas: spectral data analysis and image reconstruction and enhancement. By harnessing the power of artificial intelligence, a vast amount of spectral data acquired through spectral CT can be thoroughly explored and accurately analyzed, enabling the extraction of disease-specific spectral characteristics and composition information. Through meticulous screening, training, and validation of these features, more precise disease diagnosis and prediction can be achieved. Furthermore, given the substantial volume of data involved in spectral CT imaging, employing advanced techniques such as DLR allows for improved image resolution, reduced noise levels and artifacts, as well as enhanced image contrast.

The integration of AI and spectral CT holds significant implications for enhancing the accuracy of diagnosis and treatment for gastrointestinal tumors. With advancements in algorithms and computing power, AI surpasses human limitations in disease discrimination. The luminal structure of the gastrointestinal tract poses a constraint on human observation. In spectral imaging, where multiple parameters and subtle differences are present, energy data may not be discernible to humans but can be effectively learned by AI models to identify tumor boundaries, invasion patterns, and other intricate features with greater precision than humans.

### Multimodal imaging

Individual imaging modalities are limited in their ability to collect comprehensive medical information. For instance, CT and X-ray images provide anatomical and rigid structure information, while MRI images offer structural details along with some functional insights. On the other hand, single photon emission computed tomography (SPECT) and functional-MRI images primarily reveal detailed functional information. Due to these limitations, medical experts often face challenges in diagnosing diseases when relying solely on a single imaging modality. Consequently, the concept of multimodal imaging has been introduced to address this issue. Traditional multimodality involves integrating multiple modalities through fusion algorithms to generate detailed synthetic images. Common combinations include CT-MRI, MRI-SPECT, MRI-PET, X-ray-US, and MRI-US (Xu et al., 2020).

Spectral CT holds significant potential for multimodal imaging of gastrointestinal tumors. It represents a novel form of CT technology that not only provides structural information like traditional CT, but also enables analysis of X-ray beams with varying energies to acquire valuable insights into tumor blood supply, angiogenesis, and metabolism. By leveraging spectral CT, the limitations associated with single structure imaging in conventional CT can be overcome, thereby achieving comprehensive multimodal imaging of gastrointestinal tumors. Furthermore, when combined with other imaging modalities such as SPECT, MRI, PET, US, etc., spectral CT can offer a more comprehensive and accurate assessment of gastrointestinal tumors by complementing and validating information from different imaging techniques to enhance the precision and reliability of diagnostic results.

Besides, advances in multimodal imaging have paralleled advances in multimodal probes, especially activable multimodal imaging probes that can produce concurrent switching of signals from different imaging modalities when interacting with molecular targets. For instance, upconversion nanoparticles doped with lanthanide elements can be utilized to create a straightforward multimodal imaging probe with enhanced image quality by adjusting the Lu3+ content, which can significantly enhance contrast performance imaging in both *in vitro* upconversion luminescence and *in vivo* CT (Liu et al., 2018). Using multimodal probes, multimodal imaging of the gastrointestinal tract can also be achieved, which is very promising for gastrointestinal tumor imaging.

## Conclusion

The high incidence of GI tumors has attracted intensive attention worldwide. For the diagnosis and treatment of GI tumors, early identification of small cancers, clarifying and staging the lesions and their boundaries, assessing preoperative lymph node metastasis, and evaluating the efficacy of postoperative treatments are the most preferential concerns but remain unsolved. Endoscopy is the most common and popular diagnosis method for the gastrointestinal tract. However, its limited visual range and invasiveness remain to be replaced by improved examination methods. Notably, spectral CT provides one effective imaging method for GI tumors, and its multi-parameter imaging and quantitative analysis serve as essential references for diagnosis and treatment. Mono-energy images and basal material quantification are the most widely used means. Previous studies suggested that the unique cavity structure of the gastrointestinal tract and different imaging systems of spectral CT resulted in remarkable differences in the same type of gastrointestinal tumors. Although volume quantitative analysis is considered to be an easy way to obtain the lesion volume (Zhong et al., 2023), in most deep learning and radiomics combined with spectral CT studies, the extraction of ROI is still limited to two-dimensional level. The limitations in the application of spectral CT in GI tumor diagnosis, with the emergence of a new generation of CT technology, it is only a matter of time before solving these problems. In recent years, metabolic imaging and targeted therapy have become a focus for GI cancer profiling. Thus, molecular imaging probes and multimodal imaging of spectral CT are newly emerging urgent needs, which also can help the early detection of small tumors in the gastrointestinal tract. Furthermore, artificial intelligence is another future direction for spectra CT imaging of a huge data volume. With a powerful algorithm model, the boundaries around tumors can be accurately distinguished, and three-dimensional image features can be quickly extracted, which may be essential for evaluating invasion degrees, classification, and differentiation of gastrointestinal tumors. We firmly believe that through the continuous advancement of spectral CT, molecular imaging, and artificial intelligence, computed tomography (CT) has the potential to provide comprehensive and precise diagnosis for gastrointestinal cancer while accurately assessing treatment response and predicting prognosis.
